# Vortex formation ratio in heart failure compared to healthy volunteers at rest and during exercise

**DOI:** 10.1186/1532-429X-15-S1-O65

**Published:** 2013-01-30

**Authors:** Mikael Kanski, Johannes Toger, Katarina Steding-Ehrenborg, Marcus Carlsson, Einar Heiberg, Hakan Arheden

**Affiliations:** 1 Skåne University Hospital, Lund, Lund, Sweden

## Background

Heart failure is associated with high mortality, and diagnosis lacks reliable quantitative measures. Therefore, new measures of heart failure are needed. Vortex formation ratio (VFR) [1] is a method to describe the optimal vortex blood flow during rapid filling of the left ventricle (LV) of the heart. VFR has previously been determined using echocardiography [2] and has been proposed as a new measure of heart failure. Since magnetic resonance imaging (MRI) is the gold standard for measuring LV volumes, MRI may give more accurate VFR measurements than echocardiography. Furthermore, it is unknown if VFR is preserved during physical exercise due to the larger inflowing blood volume. Therefore, the aim was to assess VFR using MRI in heart failure and in healthy volunteers at rest and during exercise.

## Methods

Eight healthy volunteers and 14 heart failure patients (dilated cardiomyopathy. DCM) underwent cardiac MRI using a 1.5T scanner (Philips, Best, the Netherlands). Healthy volunteers were examined at rest and during physical exercise using an ergometer. LV end-systolic volume (ESV) and the blood volume before atrial contraction (diastatic volume, DV) were measured by manual delineation. E-wave volume (EWV) was defined as EWV=DV-ESV. The mean mitral valve diameter (Dm) was defined as the mean between two measurements taken from the 3-chamber long-axis view (D1) and the short-axis view of the LV (D2). The formula for calculating VFR was derived from earlier studies [2]:

VFR=4/π×EWV/Dm^3

## Results

**Figure 1:**
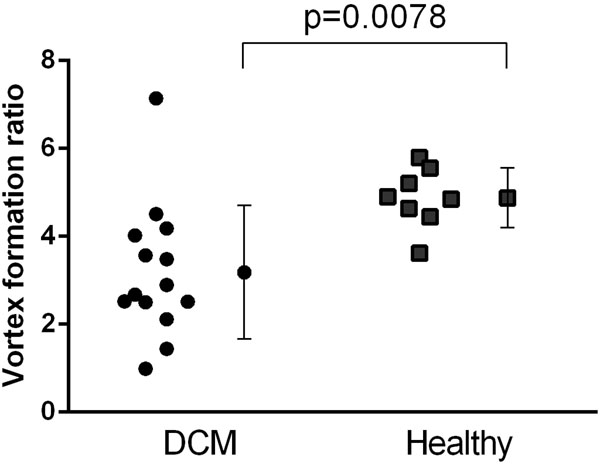
VFR in DCM patients (circles) and healthy volunteers (boxes). VFR was significantly lower in DCM compared to healthy volunteers. Error bars show mean±SD.

**Figure 2:**
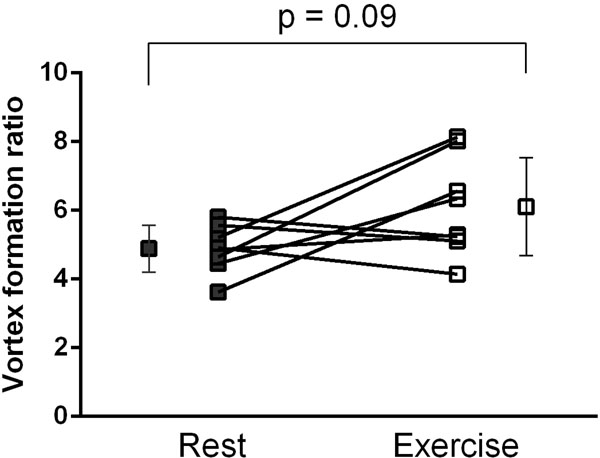
VFR in healthy volunteers at rest (closed boxes) and during exercise (open boxes). There was a non-significant trend towards slightly increased VFR during exercise. Error bars show mean±SD.

VFR was significantly lower in DCM compared to healthy volunteers at rest (figure 1: 3.2±1.5 vs 4.9±0.7, p=0.0078). In healthy volunteers, the VFR did not change significantly at exercise (figure 2: 4.9±0.7 vs 6.1±1.4, p=0.09). One patient presented with VFR=7.1. Apart from this outlier, 10/13 (77%) of the patients had VFR values lower than the lowest value in the healthy group.

## Conclusions

This study is the first to assess VFR using MRI, and to study VFR during physical exercise. DCM patients has, as a group, a significantly lower VFR than healthy volunteers which suggests that VFR can be used as a measure of heart failure. During exercise in this preliminary study, VFR did not change significantly, suggesting that optimal vortex formation is maintained at higher cardiac output.

## Funding

The Swedish Research Council; the Medical Faculty at Lund University, Sweden; the Region of Scania, Sweden; and the Swedish Heart-Lung Foundation.
